# Increasing the pneumonia treatment coverage among children under 5 years old through ‘Enhanced Management of Pneumonia in the Community’: implementation research protocol

**DOI:** 10.1136/bmjopen-2025-101346

**Published:** 2025-12-10

**Authors:** Humphreys Nsona, Emmanuel Golombe, Ulemu Zulu, Cathy Magombo, Haxson Twaibu, Prosper Mbemba, Chigomezgo Msowoya, Esau Chagoma, Aljanat Sadala, Clifford Dedza, Anusa Mangwilisa, Rudolf Banda, Nyson Chizani, Whyte Mpezeni, Shamim Ahmad Qazi, Yasir Bin Nisar

**Affiliations:** 1 IMCI Unit, Government of Malawi Ministry of Health, Lilongwe, Central Region, Malawi; 2 Kasungu District Hospital, Kasungu, Malawi; 3 H and K Data Management Centre, Lilongwe, Malawi; 4 Kamuzu Central Hospital, Lilongwe, Central Region, Malawi; 5 Independent Consultant Paediatrician, Geneva, Switzerland; 6 Department of Sexual, Reproductive, Maternal, Child and Adolescent Health and Ageing, World Health Organization, Geneva, Switzerland

**Keywords:** PAEDIATRICS, Community child health, Paediatric infectious disease & immunisation

## Abstract

**Abstract:**

**Introduction:**

Pneumonia remains a leading cause of under-5 mortality in sub-Saharan Africa, accounting for approximately 14% of deaths in this age group. In Malawi, pneumonia accounts for 12% of under-5 deaths, with recent data revealing a concerning trend of over 110 000 new cases reported in 6 months. The Malawi government has made significant strides in reducing childhood mortality through the Integrated Community Case Management (iCCM) strategy, resulting in an 11% reduction in under-5 mortality over a 5-year period. However, the current iCCM strategy does not include the management of chest indrawing pneumonia in children aged 2–59 months and fast-breathing pneumonia in infants aged up to 2 months. This implementation research aims to increase pneumonia treatment coverage for under-5 year-old children in Kasungu District, Malawi, by expanding the community-based management of pneumonia by the iCCM-trained Health Surveillance Assistants (HSAs).

**Methods and analysis:**

The current implementation research using both qualitative and quantitative data collection methods will assess the feasibility and acceptability of iCCM-trained HSAs managing chest indrawing pneumonia and fast-breathing pneumonia in children under 5 with oral amoxicillin at the community level in district Kasungu using the existing district health system. The study will employ a district health system model, leveraging existing trained iCCM HSAs to enrol and manage infants aged 7–59 days with fast-breathing pneumonia and 2–59-month-old children with chest indrawing pneumonia in the community with 7-day and 5-day oral amoxicillin, respectively. HSAs will also use pulse oximetry to identify hypoxaemic children for prompt referral to a hospital for further care. Sociodemographic features of enrolled children will be documented. Enrolled children will be followed up on treatment compliance using follow-up forms. The pneumonia treatment coverage will be assessed using baseline, midline and end-line surveys using both qualitative and quantitative data collection methods.

**Ethical and dissemination:**

Ethical approval was obtained from the National Health Research Sciences Committee and the WHO Ethics Committee. The implementation research findings will be disseminated to national-level stakeholders and specifically targeted at District Health Offices, which are responsible for implementing the interventions.

**STRENGTHS AND LIMITATIONS OF THIS STUDY**

- The integration of the study within the existing health system and the involvement of Health Surveillance Assistants enhance its potential for sustainability and scalability.
- The study employs a mixed-methods approach, combining quantitative and qualitative data to gain a comprehensive understanding of pneumonia treatment coverage and community experiences.
- Interviews with key informants among healthcare providers will help identify challenges and opportunities for improving pneumonia treatment coverage.
- Biases such as sampling bias, social desirability bias and recall bias may affect the accuracy of the findings.
- The findings may be limited by the study’s focus on a single district (Kasungu) in Malawi, possibly missing broader contextual factors influencing pneumonia treatment coverage.

## Introduction

 During the last two decades, the global under-5 mortality rate has declined to 4.9 million deaths in 2022, by nearly half compared with 2000.^1^ While this progress highlights the impact of global efforts to reduce child mortality, disparities persist. Notably, since the year 2000, neonatal deaths (within the first 28 days) decreased by 46%, compared with a 57% reduction in deaths among children aged 1–59 months. Sub-Saharan Africa, which accounts for only 30% of global live births, bears a disproportionate burden of these deaths in the same period, underscoring the need for targeted interventions to address regional disparities.^1^ Pneumonia remains a major contributor to these deaths, especially in low-income and middle-income countries.^2^ In Malawi, pneumonia is responsible for 12% of deaths in children under 5, with recent data showing more than 110 000 new cases reported within 6 months.^3^ The government has made notable progress in reducing childhood mortality through the Integrated Community Case Management (iCCM) strategy, resulting in an 11% decrease in under-5 mortality over the past 5 years.^3 4^

According to the most recent estimates, Malawi’s infant mortality rate stands at 35 deaths per 1000 live births.^1^ This rate highlights the vulnerability of newborns and the need for improved healthcare services, while the under-5 mortality rate is 48 deaths per 1000 live births, indicating a considerable risk of death for children in this age group. Malawi has made progress in reducing maternal mortality, but the ratio remains high at 225 deaths per 100 000 people.^1^ The crude death rate in Malawi is approximately 6.93 deaths per 1000 people,^1^ with various factors contributing to this rate, including infectious diseases and non-communicable diseases. The World Health Organization (WHO) classifies pneumonia by severity, a crucial predictor of mortality. Pneumonia is diagnosed by fast breathing (respiratory rate ≥50 breaths per minute in infants aged 2–11 months and ≥40 breaths per minute in children aged 12–59 months) or lower chest wall indrawing, while severe pneumonia is marked by presence of general danger signs (such as the inability to drink or breastfeed, persistent vomiting, convulsions, lethargy, unconsciousness), or stridor in a calm child, or oxygen saturation (SpO_2_) below 90%.^5^ Over 10% of pneumonia cases progress to severe disease.^6^ Given the variability in clinical skills for pneumonia classification, pulse oximetry has proven to be a valuable tool for identifying hypoxaemia, thus facilitating the detection of severe pneumonia and enabling timely interventions, thereby improving outcomes in affected children.^7^

Malawi’s iCCM component, launched in 2008, aimed to increase access to prompt treatment for pneumonia (with oral amoxicillin), diarrhoea and malaria among children under 5 years of age in hard-to-reach areas and contributed to reducing childhood mortality.^8^ The under-5 mortality rate declined from 63 deaths per 1000 live births in 2016 to 56 deaths per 1000 live births in 2021, representing an 11% reduction over 5 years.^3 4^ The success of this approach can be attributed to continuous medicine supply, supportive supervision and community acceptance. By 2021, the strategy envisioned universal coverage of treatment for these illnesses by trained and resourced Health Surveillance Assistants (HSAs), supported by their communities.^8^ However, the current iCCM protocol does not include the management of chest indrawing pneumonia in children aged 2–59 months and fast-breathing pneumonia in infants 7-59 days old, which are routinely managed at first-level health facilities by trained health professionals.^9^ Recent evidence has shown that community-level health workers like HSAs can manage these conditions at the community level,^10 11^ potentially increasing access to pneumonia treatment; however, it has not been implemented in a real-life programme community setting.

The Ministry of Health, Government of Malawi, identified Kasungu District for an implementation study aimed at increasing pneumonia treatment coverage among children under 5 by up to 80%. This study will investigate the feasibility and acceptability of scaling up the management of chest indrawing pneumonia in children aged 2–59 months and fast-breathing pneumonia in infants 7-59 days old through HSAs in the Malawian district health system (DHS).

In Malawi’s health system, the DHS model is used to strengthen health service delivery at the district level. The DHS model for Malawi provides some autonomy to districts, but it is not entirely autonomous. Districts still rely on the central government for funding, technical support and policy guidance. However, the DHS model allows districts to make decisions about resource allocation, service delivery and programme implementation.^12^ The Malawi DHS model does not generate all its resources. Instead, districts rely on a combination of funding sources, including central government funding to support health services, funding from international donors and partners to support specific health programmes and user fees from some designated patient care services to generate revenue.^12 13^ User fees are a minor source of funding in Malawi’s health system.

### Primary objective

To increase the coverage of pneumonia treatment in children under 5 years of age by up to 80% in the Kasungu district by scaling up the management of chest indrawing pneumonia in children aged 2–59 months and fast-breathing pneumonia in infants 7–59 days old through HSAs at the community level.

### Secondary objectives

(1) To evaluate the feasibility of whether iCCM-trained HSAs can manage chest indrawing pneumonia for children 2–59 months and fast breathing pneumonia among young infants aged 7–59 days in the community with oral amoxicillin for 5 and 7 days, respectively; (2) to assess treatment adherence among children treated by iCCM-trained HSAs in the community with oral amoxicillin given for 5–7 days; (3) to identify facilitating factors, barriers to implementation and challenges associated with the introduction of enhanced community case management of pneumonia among iCCM trained HSAs; (4) to determine the feasibility of using a pulse oximeter by HSAs and the acceptability of its use by HSAs and caretakers of children; (5) to identify the accuracy of fingertip pulse oximetry when used by HSAs against a standardised measurement by a trained supervisor and (6) to determine the impact of pulse oximetry on referral and treatment outcomes when combined with increased scope for HSAs to treat children.

### Methods and analysis

### Study design

Given the objectives of the implementation research, the study proposes to employ a convergent mixed-method design, where both qualitative and quantitative data will be collected concurrently, analysed separately, and then merged to provide a comprehensive understanding of the feasibility and acceptability of scaling up pneumonia management interventions. This design will enable us to triangulate findings, validate results and gain a deeper insight into the implementation process, challenges and outcomes.

We will collect quantitative data through surveys and data collection instruments. In contrast, qualitative data will be collected through key informant interviews (KIIs), focus group discussions (FGDs), at baseline, midline and end-line surveys. The integration of data from both methods will occur during the analysis phase, where we will merge and compare findings to identify patterns, themes and insights that provide a comprehensive understanding of the research questions.

We will integrate data from both methods through data transformation, where quantitative data will be summarised into thematic areas aligning with qualitative themes; triangulation, where findings will be compared and contrasted to validate results and identify areas of convergence and divergence; and data merging, creating a comprehensive dataset capturing the breadth and depth of the research questions.

### Patient and public involvement

The development of the research question was informed by the high rates of pneumonia-related morbidity and mortality. Patients were neither advisers in this study nor were they involved in the design, recruitment or conduct of the study. Results of this study will be made publicly available through open-access publication.

### Study setting, Kasungu District Hospital Structure

**Figure 1:**
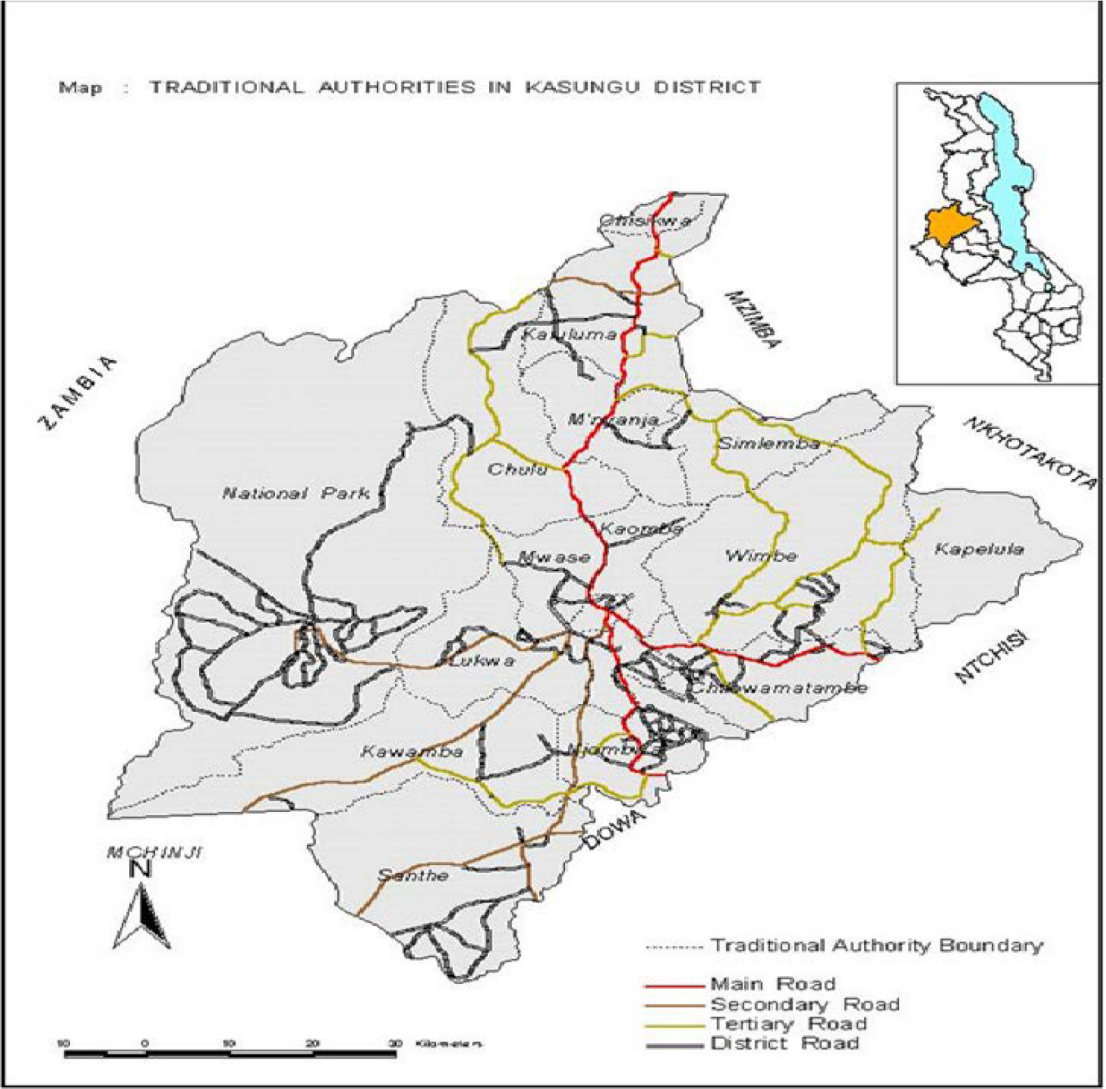
Map of Kasungu: showing location in Malawi.

Kasungu District is 1 of the 29 Districts in Malawi operating under a decentralised system located in the Central Region of the Republic of Malawi^13^ (figure 1). The population of Kasungu district has doubled from 480 659 in 1978 to 952 440 at the beginning of the implementation research in 2022. Therefore, there is a need for a corresponding increase in funding for the health sector to address the population’s health needs. About 5% of Kasungu’s population are infants aged less than 1 year, 23% are children under 5 years of age, and about 46% are aged 18 years and above.^13^

This study will be led by the Kasungu District Hospital (KDH) Health Management team members, namely the director of health and social services, district medical officer, senior medical officer, district focal persons, paediatric clinical officers, nurses, health facility in-charges and HSAs, with guidance from the Ministry of Health (MoH) national focal person for Integrated Management of Childhood Illness (IMCI) in the Ministry. Initial orientation and planning meetings will be conducted with the District Health management Team (DHMT) to enable them to conduct the study.

Kasungu District Health Office is headed by the Director of Health and Social Services, who leads the DHMT at the district level. The DHMT ensures that services are delivered according to the vision and mission of the Ministry of Health’s health sector strategic plan III.^13 14^ It provides curative, rehabilitative and preventive health services through primary and secondary levels of care. The Kisangu district hospital (KDH) is a referral facility for both health centres and rural hospitals, which also serves the local town population of 116 500, offering both inpatient and outpatient services. The inpatient services have a 350-bed capacity. KDH refers its patients to Kamuzu Central Hospital, a tertiary-level healthcare facility in Lilongwe, the capital city, which is 127 km away.^14^

As a decision-making authority, healthcare services are integrated at the primary care level and further extend to community structures through the integrated community health services delivery model. At the base of the district healthcare system are the 245 village clinics, which ensure the availability and equitable access to community-based healthcare services through village clinics.^14^ These clinics serve as the first contact points of care for sick children aged 2–59 months, operating under the iCCM strategy run by trained HSAs. The district has 38 registered health facilities, managed and headed by either a medical assistant or a clinical officer. These facilities serve and support village clinics in the following ways: (a) receive referrals from village clinics, (b) act as central hubs for storing medicines, vaccines and supplies and (c) provide clinical mentorship and supervision to village clinics and their senior HSAs.

Kasungu also implements the integrated community health services delivery model of the MoH. The programmes are integrated during service provision at households, village clinics, health posts and outreach. These programmes include Community-Based Maternal and Newborn Care, iCCM, tuberculosis, HIV, Water, Sanitation and Hygiene, Family Planning, Expanded Programme of Immunisation, Nutrition and Child Protection.^13^

**Figure 2:**
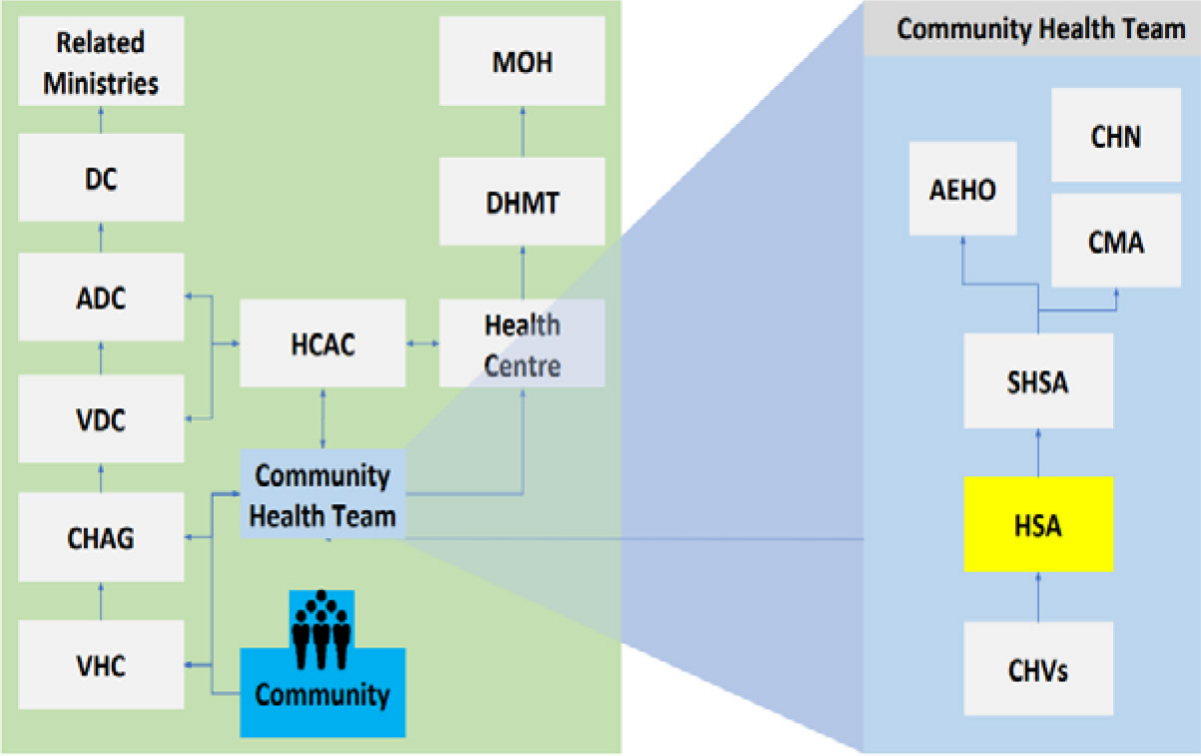
Community Health Context in Malawi. ADC, Area Development Committee; AEHO, Assistant Environmental Health Officer; CHAG, Community Health Action Group; CHN, Community Health Nurse; CHVs, Community Health Volunteers; CMA, Community Midwife Assistant; DC, District Council; DHMT, District Health Management Team; HCAC, Health Centre Advisory Committee; HSA, Health Surveillance Assistant; MoH, Ministry of Health; SHSA, Senior Health Surveillance Assistant; VDC, Village Development Committee; VHC, Village Health Committee.

As shown in figure 2, the district has various community health structures like Village Health Committees (VHCs), Community Health Action Groups (CHAG) and Health Centre Management Committees (HCMC), which meet monthly to support community health activities. These structures play an important role in the functionality of health services by mobilising resources, demand creation, as well as monitoring the delivery of quality services at the community level.^15^

### Community engagement

Community engagement offers a platform for service providers and users to interact, review service performance and address challenges in healthcare access. The district has enhanced community engagement and ownership of health services by forming and training various community health structures, such as VHCs; CHAG and HCMC. The structure committee meets monthly to support community health activities, mobilise resources, create demand and monitor the delivery of quality services. Community engagement platforms facilitate interactions between service providers and users to review service performance, explore challenges in accessing healthcare and identify solutions.^15^

This study will engage the community through various mechanisms. Initially, we will conduct sensitisation meetings to introduce the interventions for treating fast breathing and chest indrawing pneumonia under the iCCM programme, targeting children aged 2–59 months. These meetings will ensure community awareness and understanding of the study’s objectives and benefits. Additionally, we will engage the community through FGDs to gather insights and feedback during the implementation phase. Finally, we will hold dissemination meetings at the end of the study to share findings and recommendations with the community and other stakeholders. While our approach may not be fully participatory in the design phase, these engagement strategies will ensure that the community is informed, involved and invested in the study’s success.

### iCCM in Kasungu

Kasungu has 635 HSAs to work in hard-to-reach areas, of which 245 are iCCM trained, 203 reside in their respective areas and serve an estimated 45 000 under-5 children. Each HSA runs one village clinic, mainly caring for pneumonia, malaria and diarrhoeal diseases.^13^ The HSAs have two scheduled days per week to operate the village clinics. However, sick children are attended to any day.

The training of all HSAs is crucial for the successful implementation of the pneumonia management intervention. However, the study aims to assess the feasibility and acceptability of scaling up pneumonia management through HSAs in a real-world setting. The training of the remaining HSAs will occur after the study’s results are obtained as part of the plan for broader implementation and scale-up. The study’s findings will inform the training approach and facilitate the decision to equip all HSAs to manage pneumonia effectively and adequately.

### Enrolment

Young infants aged 7–59 days and children aged 2–59 months will be enrolled and tracked using screening, enrolment, sociodemographic, treatment compliance and follow-up data, as well as serious adverse event case report forms (CRFs). Newborns from birth to up to 6 days old will be referred to the nearest health facility for care, as HSAs typically do not provide treatment to this age group, focusing instead on children 7 days up to 59 months of age.

### Study timeline

The study will span 18 months, with potential extension, and will be divided into preparatory and implementation phases. The first 3 months will focus on orientation, study team formation, planning meetings and training. Data collection will occur from month 4 to month 18, accompanied by ongoing activities such as case validation visits, supervision, data review and follow-ups to ensure thorough implementation and data quality. Following data collection, the study will proceed with data analysis and subsequently disseminate the findings to stakeholders, including the community leaders.

While this study will focus on the Kasungu district, we recognise that contextual factors such as healthcare infrastructure, community demographics and socioeconomic conditions may vary across districts. To inform future scaling up, we will plan to thoroughly document and analyse these contextual factors in Kasungu, including the district’s healthcare system strengths and weaknesses, community engagement and existing health programmes, aiming to provide adaptable insights for similar settings and facilitate effective implementation and scale-up of pneumonia management interventions in districts with comparable contexts.

### Monitoring

In addition to tracking enrolled children, progress will be measured using cross-sectional surveys, including baseline, midline and endline surveys. These will use both qualitative and quantitative data collection methods. Access to pneumonia treatment, the ability of HSAs to treat young infants aged 7–59 days with fast breathing pneumonia and children aged 2–59 months with chest indrawing pneumonia at the village clinic level, and other key indicators on coverage will be compared over time to assess the success of the implementation.

The surveys will apply mixed sampling techniques. The selection of health centres and villages will apply convenience cluster-simple random sampling, while the selection of households will apply systematic random techniques.

### Household surveys

According to unpublished district reports, the prevalence of pneumonia among children under 5 years old in Kasungu district was approximately 3.5% over the past 2 weeks. Additionally, these reports indicate that about 25% of under-5 children seek appropriate care for acute respiratory illness. An increase in pneumonia treatment coverage of up to 80% is anticipated following the implementation of a district delivery model. The estimated sample size for children with pneumonia is 120, accounting for potential non-response and assuming 80% treatment coverage at both baseline and endline surveys. Based on a prevalence rate of 3.5%, the total number of children to be surveyed is projected to be 3450. Considering that 16% of the population comprises children under 5 (according to census data) and the average household consists of 4.5–5 individuals (DHS Malawi 2024), the required number of households is 4792 for both surveys. These households will be selected using probability proportional to size sampling methods^16^ from the designated study area served by the HSAs.

On survey administration, trained enumerators will administer the semistructured questionnaires to the selected households, collecting data on demographics, healthcare access and pneumonia treatment among under-5 children.

### Focus group discussions 

To gather in-depth, qualitative insights, FGDs will be conducted with mothers and caretakers of under-5 children (table 1). These discussions will explore knowledge and recognition of pneumonia signs and symptoms, treatment-seeking behaviour and decision-making processes, barriers and facilitators to accessing healthcare services, experiences with healthcare providers and facilities, and suggestions for improving pneumonia diagnosis and treatment.

**Table 1:** Sample sizes: focus group discussions and KII

Cluster name	Health facility catchment area	Number of focus group discussion mothers	KII (mothers and healthcare workers)
Kasungu District Office	Kasungu District Office		Senior medical officer (one)
Malepera	Linyangwa	1	Mother, family member, community member (three)
	Kawamba	1	
Santhe	Anchor Farm	1	Health centre in-charge HSAs managing village clinics (two)
	Santhe	1	
Nkanakhothi	Simlemba	1	Mother, family member, community member (three)
	Office	1	
Mtunthama	Chinyama	1	Health centre in-charge (one) HSAs managing village clinics (two)
	Chambwe	1	
	Totals	8	Six health workers Five mothers/caretakers

HSAs, Health Surveillance Assistants; KII, key informant interviews.

Eight FGDs will be conducted, each comprising 8–12 participants. Participants will be purposively selected to ensure diversity in age, education and socioeconomic status. FGDs will be facilitated by trained moderators using a guided discussion protocol, and discussions will be audio-recorded, transcribed and translated for analysis.

The primary objectives of the FGD are to understand mothers’ perceptions and experiences related to pneumonia diagnosis and treatment, identify knowledge gaps and treatment-seeking behaviours, and gather recommendations for improving healthcare services and community-based interventions.

Thematic analysis will be used to identify key themes and patterns, coding and categorisation will be done using qualitative data analysis software, and findings will be triangulated with quantitative data to provide a comprehensive understanding of pneumonia treatment coverage and healthcare-seeking behaviour.

### Key informant interviews

To gather nuanced insights from knowledgeable stakeholders, 11 KIIs will be conducted with 6 healthcare professionals working at selected health centres and 5 mothers/caretakers who are caregivers of under-5 children from surrounding villages (table 1).

The objectives of the KIIs are to explore health providers’ perspectives on pneumonia diagnosis and treatment, understand mothers’ and communities’ perceptions of pneumonia signs, symptoms and treatment-seeking behaviour and identify barriers and facilitators to healthcare access and utilisation.

Purposive sampling will be used to select informants with relevant expertise and experience. Semistructured interview guides will be employed to ensure consistency and flexibility, and interviews will be conducted in person, audio-recorded and transcribed for analysis. While on data analysis, thematic analysis will also identify key themes and patterns, coding and categorisation will be done using qualitative data analysis software and the findings will be triangulated with quantitative data and FGD results.

The selection criteria will include health providers who have experience working in paediatric care and have familiarity with the pneumonia treatment guidelines, mothers/caretakers with recent experience caring for under-5 children, and those who are willing to share experiences. As a sampling rationale, the six health providers will provide representative views from healthcare professionals, while the five mothers/caretakers will offer diverse perspectives from community members. This sampling strategy ensures a balanced representation of healthcare professionals’ expertise and community members’ experiences.

### Training of data collectors

iCCM-trained HSAs will be trained as enumerators. A private data management firm (H&K Data Management Centre) will conduct the training, monitor and manage the implementation data. The training will equip the enumerators with the knowledge to understand the objectives of the household survey and familiarise themselves with the data collection tools and interview guides.

### Progress and debriefs

Progress updates will be held and led by health facility in-charges every 2 days in the evening after data collection for HSAs to share field experiences and resolve any issues arising from implementation. The debriefs will involve and require HSAs to submit completed forms. This activity will enable the data management team to review the questionnaire for completeness and data quality every fortnight.

### The iCCM implementation strategy

iCCM was established in Malawi in 2008.^3 8^ Currently, there is nationwide coverage as all the 29 districts have trained HSAs who provide care and treatment for under-5 children seeking care for fever as a proxy for malaria, cough and fast breathing as a proxy for pneumonia and loose stools for diarrhoea. These HSAs administer medicines for the above three childhood illnesses at village clinics in hard-to-reach areas where they are deployed to serve communities.^3 8^

HSAs are strategically deployed in hard-to-reach, community-based settings, where they manage village clinics that serve 1000–3000 residents. These clinics provide essential healthcare services, including classification, treatment and referral of young infants less than 60 days of age and common childhood illnesses with danger signs, such as malaria, pneumonia or diarrhoea. The HSAs are provided with bicycles to support the community work. Vehicles are not provided at the community level. HSAs open and run village clinics twice or thrice a week. On the other days, HSAs conduct outreach immunisation, disease surveillance and growth monitoring activities. The HSAs are supervised by senior HSAs who are their immediate supervisors.^4 8^ The health centre nurse or clinical officer mentors and monitors the work of the HSAs.

Each HSA serves a population of between 1500 and 3000 despite the recommendation of up to 1000 population due to human resource availability constraints. Service statistics and logistics data are reported through the DHIS2 reporting platform of the MoH using relevant forms used by HSAs to compile data from the village clinic registers.^8^

### Intervention package and participants

The iCCM-trained HSAs will be trained in enhanced iCCM to treat young infants 7–59 days of age with fast breathing (60 breaths per minute or more) and oxygen saturation (SpO_2_) of 90% or more and children 2–59 months of age with chest indrawing with SpO_2_ of 90% or more with oral amoxicillin dispersible tablets (DTs) (table 2).

**Table 2:** Current and proposed enhanced Integrated Community Case Management (iCCM)

Age group	Clinical signs	iCCM	
		Current	Enhanced
0–6 days of age	Fast breathing	Refer to the hospital	Refer to the hospital
	Lower chest indrawing	Refer to the hospital	Refer to the hospital
	Hypoxaemia with pulse oximetry-SpO_2_<90%	SpO_2_ is not measured	SpO_2_ will not be measured
7–59 days of age	Fast breathing	Refer to the hospital	Treat with oral amoxicillin dispersible tablets if SpO_2_ 90% or more
	Lower chest in-drawing	Refer to the hospital	Refer to the hospital
	Hypoxaemia with pulse oximetry-SpO_2_<90%	SpO_2_ is not measured	Refer to the hospital
2–59 months of age	Fast breathing	Treat with oral amoxicillin	Treat with oral amoxicillin dispersible tablets
	Lower chest in-drawing	Refer to a first-level health facility	Treat with oral amoxicillin dispersible tablets If SpO_2_ 90% or more
	Hypoxaemia with pulse oximetry-SpO_2_<90%	SpO_2_ is not measured	Refer to the hospital

SpO_2_, oxygen saturation.

### Conceptual frameworks

**Figure 3:**
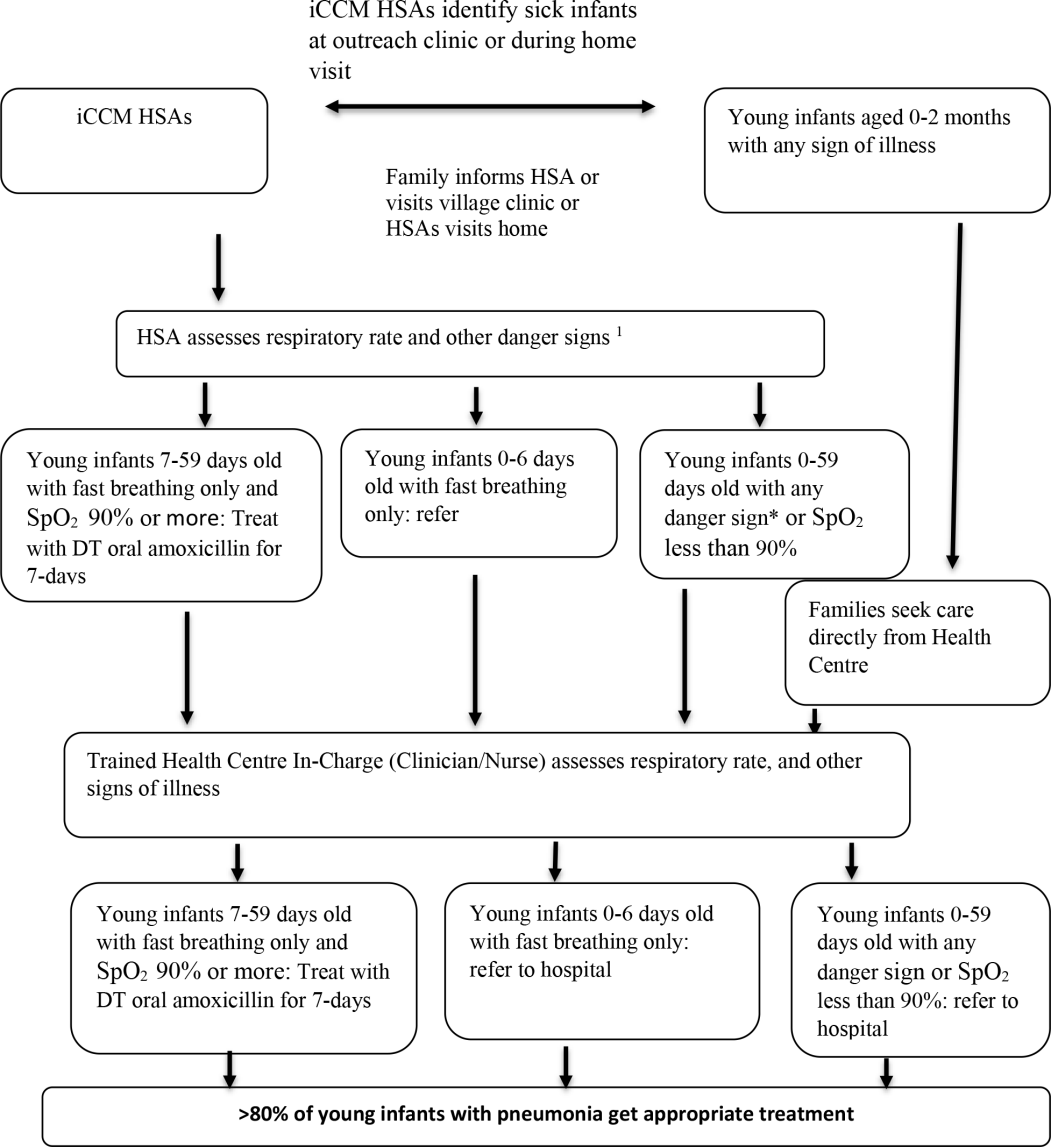
A conceptual framework for increasing coverage of pneumonia treatment in 7–59 days old young infants: (1) Danger signs in young infants <2 months of age are: not able to feed at all or stopped feeding well, convulsions or fits, movement only when stimulated or no movement at all, severe chest indrawing, high body temperature (≥38.0°C), low body temperature (<35.5°C), local infection (red or pus-draining umbilicus, skin pustules), yellow soles (jaundice), low weight (<2000 g), SpO_2_<90%. DT, dispersible tablet; HSA, Health Surveillance Assistant; iCCM, Integrated Community Case Management; SpO_2_, oxygen saturation.

**Figure 4:**
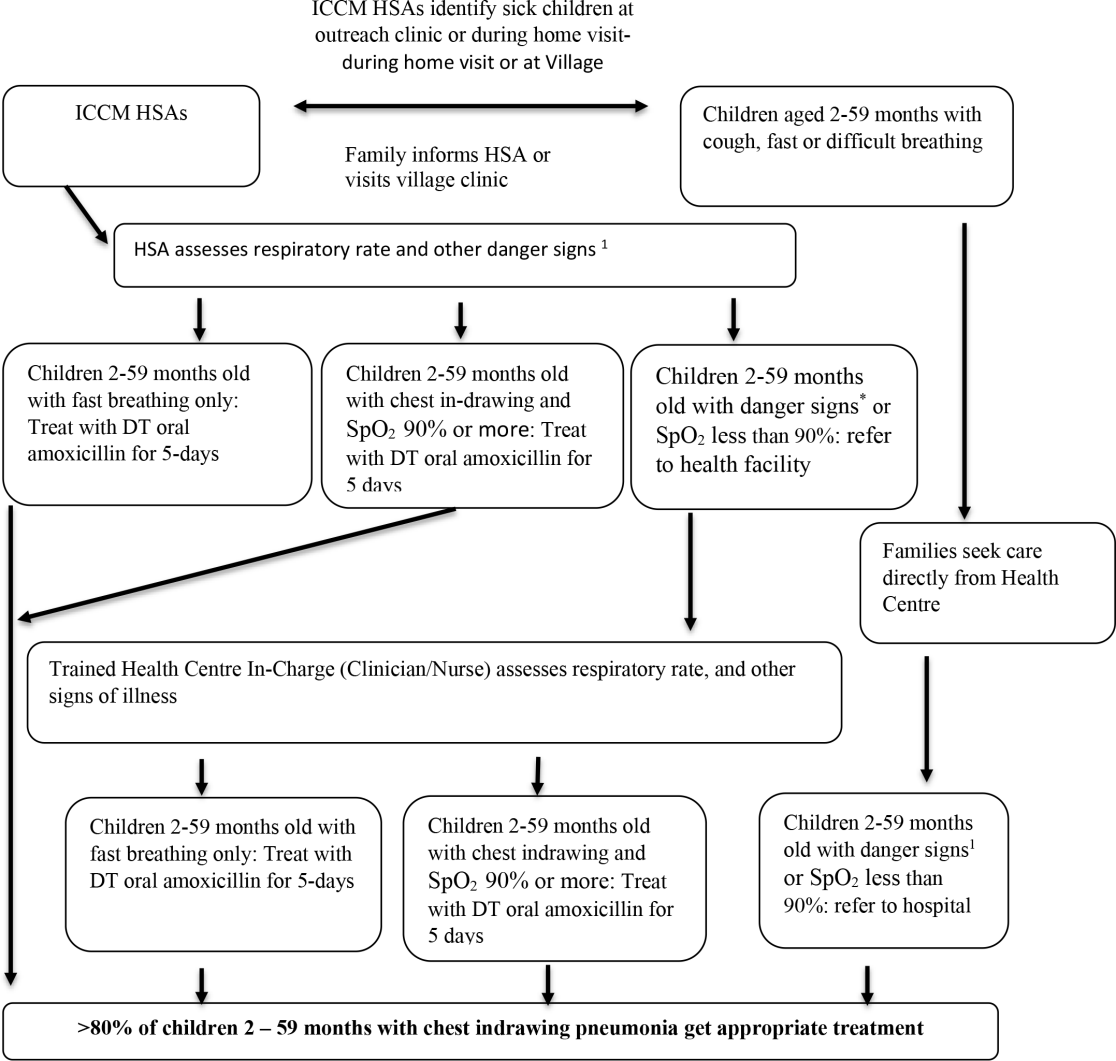
A conceptual framework for increasing coverage of pneumonia treatment in 2–59 months old children. (1) Danger signs in children 2–59 months include: Chest indrawing with SpO_2_ less than 90%, fever for 7 days or more, cough for 14 days or more, diarrhoea with blood in stool, convulsions, inability to drink or feed anything, omits everything. DT, dispersible tablet; HSA, Health Surveillance Assistant; iCCM, Integrated Community Case Management; SpO_2_, oxygen saturation.

The Malawi site had two conceptual frameworks: (1) for sick young infants aged 0–2 months (figure 3) and (2) for sick children aged 2–59 months (figure 4).

### Training, supervision, support and case confirmation

The study team will train ‘master trainers’ on an adapted version for the study of the ‘WHO/UNICEF Home Care for Newborns course for Community Health Workers,^17 18^ and the ‘study-specific procedures course’ for all study personnel. These master trainers will either be experienced clinicians or nurses considered as mentors and will then train all HSAs and service providers in the study. All supervisors and site coordinators (senior HSAs) will also be trained. Training will also be carried out using written materials, pictorial methods and iCCM video clips. Refresher training will be conducted by the master trainers.

In Malawi and Kasungu district in particular, HSAs in hard-to-reach communities are already trained in iCCM using the adapted WHO iCCM Caring for Sick Children in the Community^17^ ^18^ and they conduct an assessment, classification, decide to treat sick children on fast breathing and refer those with chest indrawing and danger signs for children 2–59 months of age.^19^ In the training for this study, knowledge and skills on the existing implementation approach will be reinforced. Furthermore, the iCCM-trained HSAs will be equipped with additional skills on how to use pulse oximeters for SpO_2_ measurement and assessment, classification and treatment of young infants. Training will vary based on the role of the study cadres. According to Malawi iCCM guidelines,^19^ all training will be conducted at the district hospitals to ensure sufficient clinical practice in outpatient clinics and inpatient wards. The details of training for different cadres will be as follows: (A) HSAs will be trained on: (1) clinical assessment (reinforcement) of infants 0–2 months and children 2–59 months according to iCCM tool, which will include measurement of body temperature, counting of respiratory rate, assessing chest indrawing and identification of danger signs. (2) Use of pulse oximetry and interpretation of SpO_2_ result. (3) Decision on which patient to refer and which patient to enrol and treat. (4) Treat children with DT oral amoxicillin according to the study protocol. (5) Counselling of caregivers on medication, feeding, general care, danger signs and when to come for unscheduled visits. (6) Completion of CRF. (B) Supervisors who, in this case, will be senior HSAs and health facility in-charges, will be trained on confirmation of enrolment, administering consent and study procedures. (C) Health centre in-charges will be trained on iCCM assessment and classification algorithm (including counting of respiratory rate, assessing chest indrawing and identification of danger signs) and case validation and clinical mentoring and (D) the district medical officer, senior medical officer and district paediatric clinical officers will serve as independent assessors and will be trained on: (1) clinical assessment of infants 0–2 months and children 2–59 months (body temperature measurement, respiratory rate counting, chest indrawing assessment and identification of danger signs), (2) use of pulse oximetry and interpretation of SpO_2_ results, (3) completion of CRF) and (4) study procedures.

Under supervision, all trained HSAs will be regularly supervised by supervisors and principal investigators to ensure the quality and continuous performance of case management.

### Case enrolments, validation, follow-up days (data tools used)

All HSAs will be trained on data collection tools, which will be adapted to the context. The study team will adapt the monthly reporting tools, form 1A and the form 1B booklet, to incorporate the sick young infant and chest indrawing variables as part of the reporting elements for DHIS2 reporting in the districts. Case enrolments will be done by enhanced iCCM-trained HSAs at the community level after the health centre in-charge (nurse or clinician) verifies the cases (table 3). The HSAs will identify cases at village clinic sites in the community they are deployed to serve.

**Table 3:** Roles and responsibilities in the study

Activity	What	By whom	When	Where	How
Identify fast breathing in sick young infants	Detection of eligible cases	HSA (Health Surveillance Assistant)	Home visits: days 1, 3 and 7 after birth Infant visits HSA at any time of day	Home village clinic	Physical and clinical assessment as per study protocol This includes measuring body temperature, counting respiratory rate, assessing chest indrawing and identifying other danger signs.
Identify chest indrawing for children aged 2 months up to 59 months	Detection of eligible cases	HSA	The child visits HSA at any time of the day	Village clinic	Physical and clinical assessment as per study protocol This includes measuring body temperature, counting respiratory rate, assessing chest indrawing and identifying other danger signs.
Screening and enrolment	Enrolling eligible cases	HSAs and HSA supervisors	When seen at home or the village clinic	Village clinic	Initial physical and clinical assessment and screening Confirmation and consent by the study supervisor (health facility in-charge) Use of pulse oximetry and interpretation of oxygen saturation results.
Treatment provision	Oral amoxicillin DT two times per day for 5 days for children 2–59 months. Oral amoxicillin DT two times per day for 7 days for young infants 7–59 days old	HSAs	On enrolment	Home village clinic	Treat children with oral amoxicillin DT according to treatment guidelines/protocol. Counselling of caretakers on medication, feeding, general care, danger signs and when to come for unscheduled visits.
Follow-up	Re-examination on enrolment and one random visit	HSAs and HSA supervisors	During follow-up visits on days 1, 2, 4, 6, 14	Home village clinic	Using case report forms (CRFs)/treatment compliance and follow-up form
Treatment documentation	Documentation of treatment using treatment compliance and follow-up form	HSAs	On enrolment	Home village clinic	Documentation, record keeping and reporting; checking CRFs completed by HSAs
Outcome assessment	Treatment failure or cured	HSAs and HSA supervisors and health centre in-charge (trained clinical officer or medical assistant)	On day 6, the HSA and HSA Supervisor will visit, and on day 14 the health centre in-charge will visit to assess the outcome	Home	Scheduled visit plan using CRF, completion of CRFs, documentation and record-keeping

DT, dispersible tablet.

Caregivers will bring their sick under-5 children to the village clinic, where the HSA will assess them for signs of pneumonia and other illnesses. Young infants identified with fast-breathing pneumonia only and sick children identified with chest indrawing pneumonia without any danger signs will be further assessed for SpO_2_. Fast-breathing pneumonia cases 7-59 days of age that will have SpO_2_ of 90% and above will be eligible for enrolment and will be treated at home with oral amoxicillin DT two times per day for 7 days, while chest indrawing pneumonia cases 2-59 months of age that will have SpO_2_ of 90% and above will be eligible for enrolment and will be treated at home with oral amoxicillin DT two times per day for 5 days. Those with SpO_2_ of less than 90% will be referred to the health facility for further management. Before enrolling an eligible case, written consent will be obtained from the caregiver for participation, as well as for follow-ups after enrolment. During the assessment, the HSAs will be using standardised enhanced iCCM CRFs and the village clinic registers.

On identifying eligible cases, the HSA will use a set of data collection tools to screen and enrol the cases. The tools used will be a screening and enrolment form, a sociodemographic form, a treatment compliance and follow-up form, and a case identification register for young infants. The in-charges will use provider observation forms to monitor the HSA’s performance during case verification.

All enrolled cases will be scheduled for follow-up on day 2, day 4, day 6 and day 14. For survival monitoring, the follow-ups will extend to day 30, day 90 and day 180. Follow-up will be conducted in two ways. Either the caregiver will bring the sick child to the village clinic for follow-up on the scheduled day, or (if the caregiver does not manage to bring the child) the HSA will visit the patients at their homes. The HSAs will use the treatment compliance and follow-up form to conduct the follow-up assessment. During follow-up, HSAs will check treatment compliance, assess the patient for danger signs and check for side effects. Throughout implementation, Senior HSAs (HSA supervisors) will routinely supervise the HSAs. The study team will also supervise the HSAs on a quarterly basis.

The study team will be conducting data validation exercises every 2 months. During data validation, the team will verify the eligibility of enrolled cases, check the completeness of data tools and conduct on-the-job coaching for health centre in-charges and Senior HSAs. The team will collect enrolment forms for all validated cases and submit them to the data management team for entry into the database.

### Pulse oximetry

SpO_2_ levels will be checked for sick young infants with fast-breathing pneumonia and sick children with chest indrawing pneumonia before enrolment (table 3) in the intervention group. Those with SpO2_2_ less than 90% will be considered to be having a danger sign and referred to a health facility. Only those with SpO2_2_ of 90% or more will be enrolled.

All implementing HSAs and health centre in-charges will be trained on how to use a pulse oximeter. Two types of pulse oximeters will be used during the implementation research, that is, fingertip pulse oximeters and Masimo Rad-5V pulse oximeters. HSAs will be trained on how to use fingertip pulse oximeters, while health centre in-charges will be trained on how to use both the Masimo Rad-5V pulse oximeter and the fingertip pulse oximeters. They will be trained on how to record the SpO_2_ in the register and data collection tools.

During the study, HSAs will use fingertip pulse oximeters to measure oxygen levels, while health centre in-charges will use Masimo Rad-5V pulse oximeters. The in-charges will be using the Masimo pulse oximeters to verify and confirm the SpO_2_ measured by the HSAs.

The district study team will ensure that the pulse oximeters are always in good working condition by replacing worn-out batteries and pulse oximeters that have stopped working.

### Referral, admission and management of sick infants and children at Kasungu District Hospital (KDH)

#### Referral of children

When a sick child is identified with severe symptoms or danger signs, they will be referred to the nearest health facility (primary healthcare centre or KDH). These facilities are typically located between 5 km and 20 km away, although the distance can vary. Referred children will be sent to the nearest health centre, where an ambulance, if availablewill be called. However, in most instances, families of referred children are responsible for arranging transportation to the district hospital, as ambulances are not always available. In Malawi, HSAs play a crucial role in delivering essential health services, providing health education and conducting disease surveillance, particularly in rural and remote areas. To qualify as HSAs, candidates must have completed at least grade 12 education.^15^ They undergo a 10-week training programme in iCCM at designated primary healthcare sites, conducted by Ministry of Health trainers. This training combines theoretical knowledge with practical experience, equipping HSAs to provide critical healthcare services effectively.^15^

All caregivers of children needing referral after assessment by the Village Clinic HSA will be counselled about the need for referral for their child. The HSAs will explain to the caregiver the need for referral and get their agreement to take the sick child to the next level of care. Following the conceptual framework that is shaped to follow the existing DHS, the next level of care is the primary care/health centre that supports the referring village clinic. In cases identified with danger signs, the village clinics will refer directly to a rural/community hospital or the district hospital. The HSAs will be taught how to calm the caregivers’ fears and help resolve any problems during the referral process, for example, fear of hospitalisation, difficulty in travelling to the hospital, etc. The HSA will prepare a referral note and a consent for follow-up at the facility, which will be endorsed/signed by the caregiver. The HSA will teach caregivers anything that is required/expected to be done on the way, such as keeping the young infant warm, breastfeeding and giving sips of Oral Rehydration Solution (ORS) if needed in case of signs of dehydration. In addition, the HSA will explain that young infants are particularly vulnerable when they are seriously ill, need hospital care and need to receive it promptly.

#### Admission and management at Kasungu District Hospital (KDH)

Where referral for in-patient care will be needed, the health centre will further refer the patient to the KDH, where the patient will be received and assessed using the WHO emergency treatment and triage approach.^20^ These cases will be admitted and managed in the paediatric wards. Cases under 30 days of age will be admitted to the neonatal care unit.

Cases that will be 30 days and above will be admitted to the paediatric high dependency unit. These cases will have access to oxygen therapy, intravenous antibiotics and further laboratory and radiology investigations to support their care.

Referred cases from study sites will be identified by receipt of an iCCM referral note, a study consent form and a sociodemographic form. Data in the wards will be captured using an inpatient follow-up form, which will record any prereferral management provided, management received during the hospital stay and treatment outcomes (discharge, referral or death).

The inpatient follow-up form will also capture all other pneumonia cases managed at the hospital other than those enrolled in the study, to appreciate the origins of these other cases as a parallel referral system and their respective outcomes.

### Supply chain, support and medicines availability

Oral amoxicillin DT used for the study will be made available through procurement support from the district medicine budget. The iCCM strategy in Malawi allows HSAs to handle and treat sick children through the availability and distribution of medicines for malaria, diarrhoea and pneumonia. In this case, trained HSAs are allowed to use anti-malarial drugs (such as artemether-lumefantrine), ORS, oral zinc supplements and oral amoxicillin DT. Oral amoxicillin DT has been available within the DHS for use by HSAs since its adoption for use by the MoH at the community level in 2013 in the country. Trained HSAs use wooden drug boxes to store medicines they obtain from the nearest health facilities to manage sick children.

During the study, a consignment of oral amoxicillin will be procured and provided from the existing Central Medical Stores system by the District Drug and Therapeutic Committee using a district supply chain procurement system. The procured medicines from the Central Medical Stores will be delivered to the District Pharmacy stores. The district pharmacist will record the medicine on stock cards as required. Thereafter, the pharmacist will update the study team on the availability of amoxicillin.

The procured medicines, including oral amoxicillin, will be distributed to health facilities. On receipt of oral amoxicillin at a health facility, the health facility in-charge will update stock cards to assess stock status levels. If the stock on hand falls below 2000 tablets, the facilities will place emergency resupply orders to restock the HSAs in the study. The district pharmacy office will resupply health facilities with amoxicillin and other medicines whenever the stock falls below the minimum level of 2000 tablets. As a routine, at the end of each month, the health facility in-charge will order medicines from the district pharmacy to restock their supply.

Similarly, from health facilities to the village clinics, at the end of every month and within 2 days of the preceding month, every iCCM-trained HSA will write a report indicating the number of cases seen in that month, the number of tablets dispensed and the number of tablets remaining (stock-on-hand).

Based on their monthly consumption and their current stock on hand, the HSAs will be resupplied with oral amoxicillin. If oral amoxicillin is stocked out at the HSA level within the month, HSAs will make an emergency order. HSAs will record stock-on-hand at the bottom of the summary page in the village clinic register, as well as on the monthly reporting form. Monitoring of medicine use will be done through supervision every month by the facility in-charge and the programme focal persons from the district level.

In Malawi’s iCCM system, the HSAs operating the village clinics in the community represent the lowest level of reporting. Every month, HSAs under each health facility submit their reports (form 1A), which are then consolidated into a single aggregate report (form 1B) for each facility, summarising the services provided and supplies used during the month.

To ensure accurate and reliable reporting, healthcare providers are encouraged to convene at the facility by the fifth of the following month to compile their reports (forms 1A and 1B) through a peer review process. This collaborative approach, supplemented by regular supervision, mentorship and review meetings throughout the study, will significantly enhance data quality. The primary beneficiaries of these reports are the HSAs and the facility staff, who use the information to inform their healthcare decisions. The reports are then forwarded to the district health office, which then sends them to the health zone and the national level, respectively, for further analysis and strategic planning.

### Data management and analysis

Once verified and completed, the data forms will be filed and organised by health facility-trained in-charges. On completion of the data collection, the finished forms will be transported to the central unit in Lilongwe, where they will undergo thorough data processing and analysis.

#### Quantitative data

The cross-sectional survey data and forms tracking the management and follow-up of children with pneumonia will be entered into a computer database using EpiInfo V.7.2. A unique identifier will link all forms related to each study child, ensuring data integrity. The management team lead will train data entry clerks on navigating the EpiInfo data entry screens and supervise the data entry and cleaning process. Once data entry is complete, the quantitative data will be exported to SPSS/PC+V.25.0 for analysis. The analysis will primarily involve generating basic statistics, frequencies and tables to summarise the data.

#### Qualitative data

The transcripts from FGDs and KIIs will be typed and stored in Microsoft Word. The data will then be analysed using NVivo V.11 to identify key themes and subthemes. The analysis will focus on various issues related to pneumonia management, including caretakers’ knowledge of signs and symptoms, treatment-seeking behaviour, appropriate medication use, treatment compliance and outcome of treatment.

The research findings will be disaggregated by age group (7–59 days and 2–59 months) and child gender. The report will also feature relevant quotes from KIIs and FGDs to provide context and depth to the findings.

### Dissemination

Research findings will be disseminated to national stakeholders, district health offices, participating facilities and community leaders to foster ownership, inform planning and ensure integration into district and community health strategies, promoting evidence-based decision-making and sustainable implementation.

## Discussion

This study’s methodology is designed to evaluate the feasibility of scaling up community-based interventions to increase pneumonia treatment coverage among children under the age of 5 years in Kasungu District, Malawi. A key strength of this study would lie in its integration with the existing DHS, enabling seamless implementation and potential long-term sustainability. However, potential challenges would include ensuring a consistent medicine supply and adequate supportive supervision.

The study’s use of pulse oximetry to measure SpO_2_ will be a notable strength; it has the potential to enable accurate identification of cases with hypoxaemia, while referral and admission procedures that are aligned with the existing DHS would ensure smooth integration. Furthermore, regular monitoring and evaluation, including supervision, mentorship, review meetings and supply chain management, will ensure the availability of oral amoxicillin and data quality.

The district health study team’s ability to integrate the implementation research with the existing health system will provide the potential to generate critical evidence on the effectiveness of chest in-drawing pneumonia and fast breathing pneumonia treatment for community-based interventions in reducing childhood pneumonia mortality in Malawi. This study’s findings will contribute to the global effort to reduce under-5 mortality, aligning with the Sustainable Development Goal target 3.2.1.

## Conclusions

This implementation research study aims to address the critical gap in pneumonia treatment coverage for under-5 children in Kasungu District, Malawi, by leveraging the existing iCCM implementation platform and trained HSAs. By empowering trained HSAs to manage chest indrawing and fast-breathing pneumonia in the community using oral amoxicillin, this study has the potential to significantly reduce pneumonia-related morbidity and mortality in this vulnerable age group. The study’s findings will provide valuable insights into the feasibility and acceptability of this community-based approach, informing future scale-up and policy decisions.
