# Supplementary material for: Increasing the pneumonia treatment coverage among children under 5 years old through ‘Enhanced Management of Pneumonia in the Community’: implementation research protocol
Source: BMJ Open. 2025 Dec 10;15(12):e101346. doi: 10.1136/bmjopen-2025-101346 (PMC12699558; doi:10.1136/bmjopen-2025-101346)
Supplement: Supplementary file 1 [file bmjopen-2025-101346.reviewer_comments.pdf]

PEER REVIEW HISTORY

BMJ Open publishes all reviews undertaken for accepted manuscripts. Reviewers are asked to complete a checklist review form and are provided with free text boxes to elaborate on their assessment. These free text comments are reproduced below.

ARTICLE DETAILS

Title (Provisional)

Increasing the pneumonia treatment coverage among under five years old children through ‘Enhanced Management of Pneumonia in the Community’: Implementation Research Protocol

Authors

Nsona, Humphreys; Golombe, Emmanuel; Zulu, Ulemu; Magombo, Cathy; Twaibu, Haxson; Mbemba, Prosper; Msowoya, Chigomezgo; Chagoma, Esau; Sadala, Aljanat; Dedza, Clifford; Mangwilisa, Anusa; Banda, Rudolf; Chizani, Nyson; Mpezeni, Whyte; Qazi, Shamim; Nisar, Yasir Bin

---

VERSION 1 - REVIEW

Reviewer 1

Name Doke, Prakash

Affiliation Bharati Vidyapeeth Deemed University Medical College, Community Medicine

Date 03-May-2025

COI None

Abstract: The authors may explain the use of the term district health system model. Is it autonomous? Will it generate its own resources? All programs use the district model. Where will the child be referred? The authors may write a few lines about HSAs, their education, training, etc. The type of institute, the average distance, and the availability of vehicles.

Introduction: The word 'only 30%' may be replaced by 30%. The authors may also provide data about mortality to substantiate their claim that mortality is disproportionate.

The rationale given in the introduction and the primary objective do not match.

The script does not mention about the children 1-7 days; it is mentioned in the table.

The sample size calculation assumes that 50% of the children might have received treatment. This means the authors have to screen a large population to find 120 children with a history of pneumonia. Some attention also needs to be paid to the design effect, which may further increase the sample size.

The authors may disseminate the findings of the project to the community.

Only 245 out of 635 are trained, but the authors may ensure that all are trained.

The ICC is assumed to be 0.02 when calculating the second sample size. The authors may provide some references.

**** **The reviewer provided a marked copy with additional comments. Please contact the publisher for full details.** ****

Reviewer 2

Name Kundu, Satyajit

Affiliation Patuakhali Science and Technology University, Biochemistry and Food Analysis

Date 05-Jun-2025

COI None

The manuscript presents a well-designed implementation research protocol addressing a critical child health issue—pneumonia treatment among under-five children in Malawi. The protocol has several strengths, including the strong integration into the existing district health system, and the use of Health Surveillance Assistants (HSAs) adds potential for sustainability and scalability, employing a mixed-methods approach (quantitative + qualitative) enhances the study's comprehensiveness and validity, etc. For further clarification, I have some feedback.

- The manuscript would benefit from a clearer description of the study timeline, including start/end dates of data collection for each phase.

- Under the "Community engagement" section, it would be better to mention whether you have any plans to engage the community in your design process? It is very important to study with the community (not on the community), especially in terms of implementation research. So, how do you plan to engage the community in the design process and to implementation phase of your study? Would it be a participatory approach? If no, better to provide rationale, why? I would suggest engaging the community at least like a tokenism participation.

- For study design, the authors mentioned that "A mixed method (qualitative and quantitative) implementation research" will be employed. I would suggest to clarify for about the mixed-method design, like will it be a convergent mixed-method design or a sequential mixed-method design? Though you have described the procedures in your text, mentioning this under the "study design" section would be better for the readers. Also, it would be worth mentioning the integration process of data from both qualitative and

quantitative methods. How will you integrate data from both methods? Are you planning to do the survey first and then qualitative interviews / FGDs?

- While a design effect is applied, further justification or references for using a design effect of 2 in the household survey sample size calculation would strengthen the methodological rigour.

- The authors acknowledge limited generalizability due to the single-district design (Kasungu); however, it would be helpful to discuss potential contextual factors in more depth to inform future scaling up into other similar settings.

VERSION 1 - AUTHOR RESPONSE

Reviewer 1

C1.1: The authors may explain the use of the term district health system (DHS) model. Is it autonomous? Will it generate its own resources?

Authors' Response:

Thank you for your insightful query regarding the District Health System (DHS) model in Malawi. We have added the following statement on **page 5** of the revised manuscript:

In Malawi's health system, the District Health System (DHS) model plays an important role in enhancing health service delivery at the district level. While it provides some degree of autonomy to districts, it is not entirely self-sufficient. Districts continue to depend on the central government for essential funding, technical support, and overarching policy guidance. The DHS model empowers districts to make informed decisions about resource allocation, service delivery, and programme implementation. However, it is important to note that the DHS model does not generate all of its financial resources independently. Instead, it operates through a combination of funding sources, including central government funding to support health services, international donors and partners funding to support specific health programmes and user fees from some designated patient care services to generate revenue. User fees are a minor source of funding in Malawi's health system.

C1.2: Where will the child be referred? The authors may write a few lines about HSAs, their education, training, etc. The type of institute, the average distance, and the availability of vehicles.

Authors' Response:

Thank you for your valuable comments regarding child referrals and the role of HSAs in Malawi. We have explained it as follows and inserted on **page 17** of the revised manuscript:

When a sick child is identified with severe symptoms or danger signs, they are referred to the nearest health facility (primary healthcare centre or district hospital). These facilities are typically located between 5 km and 20 km away, although distance can vary. Referred children are sent to the nearest health centre, where an ambulance, if available, is called. However, in most instances, families of referred children are responsible for arranging transportation to the district hospital, as ambulances are not always available. In Malawi, HSAs play a crucial role in delivering essential health services, providing health education, and conducting disease surveillance, particularly in rural and remote areas. To qualify as HSAs, candidates must have completed at least grade 12 education. They undergo a 10-week training program in Integrated Community Case Management (iCCM) at designated primary healthcare sites, conducted by Ministry of

Health trainers. This training combines theoretical knowledge with practical experience, equipping HSAs to provide critical healthcare services effectively.

The below paragraph is inserted on **page 11** of the revised manauscript:

HSAs are strategically deployed in hard-to-reach community-based settings, where they manage village clinics that serve 1000-3000 residents. These clinic provides essential healthcare services, including classification, treatment, and referral of young infants less than 60 days of age and common childhood illnesses with danger signs, such as malaria, pneumonia, or diarrhoea. The HSAs are provided with bicycles to support the community work. Vehicles are not provided at the community level. HSAs open and run village clinics twice or thrice a week. On the other days, HSAs conduct outreach immunisation, disease surveillance and growth monitoring activities. The HSAs are supervised by senior HSAs who are their immediate supervisors. The health centre nurse or clinical officer mentors and monitors the work of the HSAs.

C1.3: The authors may also provide data about mortality to substantiate their claim that mortality is disproportionate.
Authors' Response:
Thank you for your suggestion to provide mortality data to support our claims regarding disproportionate mortality rates. We have added this on **page 4** of the revised manuscript, as given below:
According to the most recent estimates, Malawi's infant mortality rate stands at 35 deaths per 1,000 live births.[1] This rate highlights the vulnerability of newborns and the need for improved healthcare services. Similarly, the recent under-5 mortality rate is 48 deaths per 1,000 live births, indicating a considerable risk of death for children in this age group. Malawi has made progress in reducing maternal mortality, but the ratio remains high at 225 deaths per 100,000 people (2024 UN estimates).[2] The crude death rate in Malawi is approximately 6.93 deaths per 1,000 people (2024 DHS), with various factors contributing to this rate, including infectious diseases and non-communicable diseases.
C1.4: The rationale given in the introduction and the primary objective do not match.
Authors' Response:
Thank you for your comment. The Primary Objective has been rephrased to specify both age groups so that it matches the rationale in the introduction in the revised manuscript on **page 5**, as given below:
"To increase the coverage of pneumonia treatment in children under five years of age by up to 80% in the Kasungu district by scaling up the management of chest indrawing pneumonia in children aged 2-59 months and fast-breathing pneumonia in infants 7- 59 days old through HSAs at the community level."
C1.5: The script does not mention about the children 1-7 days; it is mentioned in the table.
Authors' Response:
We have added the following text to the manuscript and inserted the below text on **page 8** of the manuscript:
"Newborns from birth to 6 days old are referred to the nearest health facility for care, as HSAs typically do not provide treatment to this age group, focusing instead on children 7 days up to 59 months of age*"*.
C1.6: The sample size calculation assumes that 50% of the children might have received treatment. This means the authors have to screen a large population to find 120 children

---

[1] Malawi Demographic and Health Survey 2024 key indicators report: (available from:https://www.nsomalawi.mw/publications/demography/malawi-demographic-and-health- survey-2024-key-indicators-report)
[2] World Health Organization. *Trends in maternal mortality 2000 to 2020: estimates*. 2021 *WHO, Geneva* (p. 12). Retrieved from https://www.who.int/reproductivehealth/publications/maternal-mortality-2000-2017/en/

with a history of pneumonia. Some attention also needs to be paid to the design effect, which may further increase the sample size.
Authors' Response*:*

Thank you for your comments regarding the sample size calculation and the implications of the design effect. We have updated the sample size in the revised manuscript on **page 9** as shown below:

According to unpublished district reports, the prevalence of pneumonia among children under five years old in Kasungu district was approximately 3.5% over the past two weeks. Additionally, these reports indicate that about 25% of under-five children seek appropriate care for acute respiratory illness. An increase in pneumonia treatment coverage of up to 80% is anticipated following the implementation of a district delivery model. The estimated sample size for children with pneumonia is 120, accounting for potential non-response and assuming 80% treatment coverage at both baseline and endline surveys. Based on a prevalence rate of 3.5%, the total number of children to be surveyed is projected to be 3,450. Considering that 16% of the population comprises children under five (according to census data) and the average household consists of 4.5 to 5 individuals (DHS Malawi 2024), the required number of households is 4,792 for both surveys. These households will be selected using Probability Proportional to Size (PPS) sampling methods from the designated study area served by the HSAs.

C1.7: The authors may disseminate the findings of the project to the community.

Authors' Response:

Thank you very much. We have added the following to the revised manuscript on **page 20**:

Research findings will be disseminated to national stakeholders, District Health Offices, participating facilities and community leaders to foster ownership, inform planning, and ensure integration into district and community health strategies, promoting evidence-based decision-making and sustainable implementation

C1.8: Only 245 out of 635 are trained, but the authors may ensure that all are trained.

Authors' Response:

Thank you for your comment. We have included the following text on **page 8** of the manuscript:

The training of all HSAs is crucial for the successful implementation of the pneumonia management intervention. However, the current study aims to assess the feasibility and acceptability of scaling up pneumonia management through Health Service Areas (HSAs) in a real-world setting. The training of the remaining HSAs will occur after the study's results are obtained as part of the plan for broader implementation and scale-up. The study's findings will inform the training approach and facilitate the decision to equip all HSAs to manage pneumonia effectively and adequately.

C1.9: The ICC is assumed to be 0.02 when calculating the second sample size. The authors may provide some references.

Authors' Response:

Thank you very much for your comment. We agreed with the reviewer. In fact, we don't need a sample size for this as we will enrol all young infants and children who will be assessed and treated by the HSAs in the study catchment areas. Hence we have removed this sample size from the manuscript.

<u>Reviewer 2</u>

The manuscript presents a well-designed implementation research protocol addressing a critical child health issue—pneumonia treatment among under-five children in Malawi. The protocol has several strengths, including the strong integration into the existing district health system, and the use of Health Surveillance Assistants (HSAs) adds potential for sustainability and scalability, employing a mixed-methods approach (quantitative + qualitative) enhances the study's comprehensiveness and validity, etc. For further clarification.

C2.1: The manuscript would benefit from a clearer description of the study timeline, including start/end dates of data collection for each phase.

Authors' Response:

We have included the following statements on **<u>page 8</u>** of the manuscript:

The study will span 18 months, with potential extension, and will be divided into preparatory and implementation phases. The first three months will focus on orientation, study team formation, planning meetings, and training. Data collection will occur from month 4 to month 18, accompanied by ongoing activities such as case validation visits, supervision, data review, and follow-ups to ensure thorough implementation and data quality. Following data collection, the study will proceed with data analysis and subsequently disseminate the findings to stakeholders, including the community leaders.

C2.2: Under the "Community engagement" section, it would be better to mention whether you have any plans to engage the community in your design process? It is very important to study with the community (not on the community), especially in terms of implementation research. So, how do you plan to engage the community in the design process and to implementation phase of your study? Would it be a participatory approach? If no, better to provide rationale, why? I would suggest engaging the community at least like a tokenism participation.

Authors' Response:

We have added text on **<u>page 8</u>** of the manuscript under community engagement*:*

This study will engage the community through various mechanisms. Initially, we will conduct sensitisation meetings to introduce the interventions for treating fast breathing and chest indrawing pneumonia under the iCCM program, targeting children aged 2-59 months. These meetings will ensure community awareness and understanding of the study's objectives and benefits. Additionally, we will engage the community through Focus Group Discussions (FGDs) to gather insights and feedback during the implementation phase. Finally, we will hold dissemination meetings at the end of the study to share findings and recommendations with the community and other stakeholders. While our approach may not be fully participatory in the design phase, these engagement strategies will ensure that the community is informed, involved, and invested in the study's success.

C2.3: For study design, the authors mentioned that "A mixed method (qualitative and quantitative) implementation research" will be employed. I would suggest to clarify for about the mixed-method design, like will it be a convergent mixed-method design or a sequential mixed-method design? Though you have described the procedures in your text, mentioning this under the "study design" section would be better for the readers.

Authors' Response:

Thank you for your comment. We have included the following revised text on **<u>page 5</u>** of the manuscript*:*

Given the objectives of the implementation research, the study proposes to employ a convergent mixed-method design, where both qualitative and quantitative data will be collected concurrently, analysed separately, and then merged to provide a comprehensive understanding of the feasibility and acceptability of scaling up pneumonia management interventions. This design will enable us to triangulate findings, validate results, and gain a deeper insight into the implementation process, challenges, and outcomes

C2.4: Also, it would be worth mentioning the integration process of data from both qualitative and quantitative methods. How will you integrate data from both methods? Are you planning to do the survey first and then qualitative interviews / FGDs?

Authors' Response:

The paragraphs below have been inserted on **page 6** of the manuscript:

We will collect quantitative data through surveys and data collection instruments. In contrast, qualitative data will be collected through key informant interviews (KII), focus group discussions (FGDs), and observations during the baseline, midline and end-line surveys. The integration of data from both methods will occur during the analysis phase, where we will merge and compare findings to identify patterns, themes, and insights that provide a comprehensive understanding of the research questions.

We will integrate data from both methods through data transformation, where quantitative data will be summarised into thematic areas aligning with qualitative themes; triangulation, where findings will be compared and contrasted to validate results and identify areas of convergence and divergence; and data merging, creating a comprehensive dataset capturing the breadth and depth of the research questions.

C2.5: While a design effect is applied, further justification or references for using a design effect of 2 in the household survey sample size calculation would strengthen the methodological rigour.

Authors' Response:

We have addressed this by responding to Reviewer 1 comments on sample size calculations (C6 and C9) and removing the second sample size calculation from the revised manuscript. Additionally, we have revised the sample size calculations for the two surveys. Please refer to **page 9** of the revised manuscript.

C2.6: The authors acknowledge limited generalizability due to the single-district design (Kasungu); however, it would be helpful to discuss potential contextual factors in more depth to inform future scaling up into other similar settings.

Authors' Response:

The paragraph below has been inserted on **pages 8 & 9** of the manuscript:

While our study will focus on the Kasungu district, we recognise that contextual factors such as healthcare infrastructure, community demographics, and socioeconomic conditions may vary across districts. To inform future scaling up, we will plan to thoroughly document and analyse these contextual factors in Kasungu, including the district's healthcare system strengths and weaknesses, community engagement, and existing health programs, aiming to provide adaptable insights for similar settings and facilitate effective implementation and scale-up of pneumonia management interventions in districts with comparable contexts.

VERSION 2 - REVIEW

**Reviewer**	**1**
**Name**	**Doke, Prakash**
**Affiliation**	**Bharati Vidyapeeth Deemed University Medical College, Community Medicine**
**Date**	**25-Jul-2025**
**COI**	

The authors have incorporated almost all suggestions.
